# The efficacy of Chinese patent medicine combined with entecavir for the treatment of chronic HBV-related liver fibrosis or cirrhosis: Protocol for a systematic review and meta-analysis of randomized controlled trials or prospective cohort studies

**DOI:** 10.1097/MD.0000000000015732

**Published:** 2019-05-31

**Authors:** Yudi Song, Junkai Zhao, Sudan Wang, Haoming Huang, Junwei Hong, Junling Zuo, Shaochuan Huo

**Affiliations:** a The First Clinical Medical College, Guangzhou University of Chinese Medicine; b Department of Emergency, The First Affiliated Hospital of Guangzhou University of Chinese Medicine; c Southern Medical University, Guangzhou, P.R. China.

**Keywords:** Chinese patent medicines, chronic, cirrhosis, hepatitis B, liver fibrosis, network meta-analysis

## Abstract

**Background:**

There are currently no FDA-approved biological or chemical drugs for the treatment of HBV-related liver fibrosis or cirrhosis. Some Chinese patent medicines have proven to be effective in this area.

**Objective:**

The network meta-analysis (NMA) is to evaluate whether entecavir combined with Chinese patent medicine, such as “fuzhenghuayu capsules,” “anluohuaxian pills,” “fufangbiejiaruangan tablets,” shows superior efficiency compared with entecavir alone for the treatment of chronic HBV-related liver fibrosis or cirrhosis. To evaluate which Chinese patent medicine is the most effective at improving liver fibrosis or cirrhosis in chronic hepatitis B-infected patients?

**Methods:**

Registration of protocol: the protocol was published in the PROSPERO database (identification number: CRD42018112547). We will search PubMed, EMbase, Medline, Cochrane, China Network Knowledge Infrastructure (CNKI), and Wanfang for randomized controlled trials (RCTs) or “prospective cohort studies” of “fuzhenghuayu capsules,” “anluohuaxian pills,” ”fufangbiejiaruangan tablets” respectively combined with entecavir in the treatment of chronic HBV-related liver fibrosis or cirrhosis from their inception to September 30, 2018. R 3.3.3 and GeMTC 0.14.3 software will be used for data analysis.

## 1 Introduction

There is no cure for chronic hepatitis B (CHB) currently. In 2015, CHB caused 887,000 deaths, most of which were due to complications (including cirrhosis and hepatocellular carcinoma).^[1]^ The ultimate goal of CHB treatment is to delay and reduce the occurrence of liver failure, cirrhosis decompensation, liver cancer and other complications so as to improving patients’ quality of life and prolonging life.^[2]^ Hepatitis, cirrhosis, and liver cancer are called trilogy of chronic liver disease, and liver fibrosis is a stage that must be passed before CHB causes cirrhosis. Therefore, timely diagnosis and treatment of liver fibrosis is of great significance for CHB patients.

Liver fibrosis and a certain degree of cirrhosis have been confirmed to be reversible.^[3]^ Current medicine treatments of liver fibrosis or cirrhosis caused by CHB are divided into 2 aspects: etiological treatment and anti-fibrosis treatment. Etiology treatment: that is, anti-HBV treatment; specific anti-fibrosis treatment: antioxidants such as resveratrol,^[4,5]^ vitamin E, phosphatidylcholine, silymarin, and so on. They can reduce oxidation stimulation, inhibit the activation of hepatic stellate cells (HSC) and reduce liver fibrosis; colchicine,^[6]^ hepatocyte growth factor,^[7]^ etc. can increase collagenase activity and enhance collagen degradation; others: some studies have shown that vitamins D_3_ can inhibit the activation and proliferation of HSC^[8]^; 1,25-(OH)_2_D_3_ can reduce the deposition of extracellular matrix and the degree of hepatic fibrosis.^[9]^ However, the above drugs lack sufficient evidence-based medical evidence for the treatment of liver fibrosis, and the researches on some drugs only stay in the preliminary experiment stage. There is no FDA-approved biochemical drug for liver fibrosis at present.

Traditional Chinese medicine (TCM) has accumulated rich clinical experience in the treatment of CHB and CHB related complications. At present, it has formed the “Guidelines for diagnosis and treatment of liver fibrosis with integrated traditional Chinese and Western medicine”^[3]^ and “Consensus Opinions on Diagnosis and Treatment of Liver Fibrosis with Integrated Traditional Chinese and Western Medicine.”^[10]^ For example, fuzhenghuayu capsules, anluohuaxian pills, fufangbiejiaruangan tablets, dahuangzhechong pills, biejiajian pills, and qianggan capsules are all recommended Chinese patent medicines for the treatment of liver fibrosis or cirrhosis. There are the most clinical application and research about the first 3 Chinese patent medicines ^[11–15]^, but there is no comparison of the efficacy among them.^[16]^ So the author thinks it is necessary and meaningful to conduct a network meta-analysis (NMA) to compare the efficacy among the 3 Chinese patent medicines for the treatment of chronic HBV-related liver fibrosis or cirrhosis. It also can provide some reference for the clinical application of related drugs.

## 2 Methods

### 2.1 Protocol and registration

A protocol had been registered in PROSPERO CRD42018112547.

### 2.2 Criteria for included studies

Randomized controlled trials (RCTs) or prospective cohort studies with clear diagnostic criteria for hepatitis B-related liver fibrosis or cirrhosis can be included

#### 2.2.1 Types of participants

People with HBV-related liver fibrosis or cirrhosis diagnosed by validated diagnostic criteria, regardless of age, sex, or ethnic origin.

#### 2.2.2 Types of interventions

Fuzhenghuayu capsules (1.5 g tid po), anluohuaxian pills (6 g bid po), fufangbiejiaruangan tablets (2 g tid po) respectively plus entecavir (0.5 mg qd po), entecavir (0.5 mg qd po). The course of treatment should be 6 months at least.

#### 2.2.3 Outcome measures

Primary outcomes: all-cause mortality, liver biopsy, quality of life score, serious adverse reactions. Secondary outcomes: Virus markers: HBV-DNA, HBeAg; liver stiffness; liver fibrosis biomarkers (hyaluronic acid [HA], laminin [LN], type IV collagen [IV-C], and type III procollagen N-terminal peptide [PIIINP]); B-ultrasound performance: portal vein diameter, spleen vein diameter, spleen thickness; child-pugh score; liver function biomarkers (alanine aminotransferase [ALT], aspartarte aminotransferase [AST], albumin [ALB], and total bilirubin [TBiL]); general adverse reactions.

#### 2.2.4 Ethics and dissemination

The data used in this study are derived from relevant data in published academic papers. It does not require ethical approval and the results of this paper will be published in an open form in internationally influential academic journals.

### 2.3 Search strategy and study selection

#### 2.3.1 Database selection

We will search the following databases: PubMed, EMBASE, Medline, Cochrane, CNKI, Wanfang from their inception to September 30, 2018.

#### 2.3.2 Search strategy

The search terms will include the following: ”liver fibrosis,” ”cirrhosis,” “hepatitis B, Chronic,” ”fuzhenghuayu capsules,” “anluohuaxian pills,” “fufangbiejiaruangan tablets,” “randomized controlled trials (RCTs),” “prospective cohort study,” “entecavir” in English or Chinese.

### 2.4 Data collection and analysis

#### 2.4.1 Data extraction

Two review authors will extract data independently concerning details of study design, participant characteristics, interventions, and outcomes using a self-designed data extraction form. The data extraction form will include the following items: methods (trial design, sample size calculations, length of follow-up, and information needed to assess the risk of bias); participants (age, duration of chronic hepatitis B, diagnostic criteria, withdrawal/loss to follow-up, etc.); interventions (medicines, dosage, duration, etc.); outcomes, and results. We will attempt to obtain missing information by contacting authors whenever possible, and any discrepancies will be resolved by consensus.

#### 2.4.2 Assessment of risk of bias

Two people will assess the risk of bias for each included study independently following the instructions given in the Cochrane Handbook for Systematic Reviews of Interventions and the Cochrane Hepato-Biliary Group Module. These cover 7 points: random sequence generation; allocation concealment; blinding of participants and personnel; blinding of outcome assessment; incomplete outcome data; selective outcome reporting; other bias. Disagreements will be resolved by discussion or through arbitration with a third review author when necessary. The quality of all the included trials will be evaluated as being of low, unclear, or high risk of bias.

#### 2.4.3 Data synthesis

All statistical analyses will be performed using Robert Gentleman, Ross Ihaka, Douglas Bates, et al. and Gert van Valkenhoef, Sylwia Bujkiewicz, Orestis Efthimiou, et al. R software network package will be used to draw a network relationship graph of each outcome indicator, with each node representing an intervention medicine, the node size indicating its sample size, and the connection thickness between nodes indicating the number of studies included in the intervention. Consistency Hypothesis Test: node analysis will be performed by the node split method using the GeMTC software. Based on the Markov chain Monte Carlo (MCMC) algorithm, all control types that can be reasonably split (with independent indirect evidence) will be presented, and the inconsistency *P* value of each node type will be calculated. If *P* > .05, the judgment is consistent, and the result will be selected consistency model analysis. If *P* < .05, it is be judged as inconsistent, and the result will be selected consistency model analysis.^[17]^ Model goodness-of-fit test; potential scale reduction factor (PSRF) quantitative analysis will be used to diagnose the convergence degree of the model. If the PSRF value is close to 1, the evaluation model has better convergence.^[18]^ The ranking probabilities for assessing individual interventions obtained under the Bayesian network model: the cumulative ranking probability bar chart of the corresponding drugs will be plotted, and according to the area of bar chart, to judge the best efficacy of the drugs, the larger the area of bar chart, the better the efficacy. Network meta-analysis result: when the outcome indicator is count data, the odds ratio (OR) and its 95% confidence interval (CI) are used for data analysis; when the outcome indicator is measurement data, the mean difference (MD) and its 95% CI are used for data analysis.

## 3 Discussion

CHB cannot be cured at present. Most CHB patients die from its complications such as cirrhosis or liver cancer. Hepatitis, cirrhosis, liver cancer commonly known as chronic Liver disease trilogy, and hepatic fibrosis is the only way to develop hepatitis to cirrhosis. Early detection and treatment of hepatic fibrosis can delay and reduce the incidence of liver failure, decompensated cirrhosis, hepatocellular carcinoma, and other complications so as to improve patients’ life quality and prolong life. However, due to the complex pathogenesis of hepatic fibrosis, there is no FDA approved biochemical drugs for the treatment of hepatic fibrosis now. TCM does not have liver fibrosis or cirrhosis disease name. According to its clinical manifestation it can be attributed to “blood stasis,” “ lump in the abdomen,” “jaundice,” “hepatic accumulation,” and other diseases of the category.

TCM has a deep understanding of CHB and related complications accumulated a wealth of clinical treatment experience. At present, there have been lots of curative effect evaluations of anti-hepatic fibrosis of traditional Chinese medicine. Fuzhenghuayu capsules, anluohuaxian pills, and fufangbiejiaruangan tablets are all in the first line of recommended drugs in TCM guidelines or consensus of liver fibrosis. There are some meta-analysis of hepatic fibrosis or cirrhosis about the above 3 drugs in combination with entecavir.^[11–15]^ But there is no comparison of the efficacy among the above 3 drugs now. So we feel that it is necessary and meaningful to conduct a network-meta analysis to compare the efficacy of the above 3 drugs in the treatment of CHB related hepatic fibrosis or cirrhosis, it can provide some reference for clinical application of related drugs.

## Author contributions

**Data curation:** Yudi Song, Junling Zuo, Shaochuan Huo.

**Formal analysis:** Yudi Song, Haoming Huang, Junwei Hong, Junkai Zhao, Sudan Wang.

Funding acquisition: Junling Zuo.

**Methodology:** Haoming Huang, Junwei Hong.

**Project administration:** Junling Zuo.

**Software:** Junwei Hong, Junkai Zhao.

**Supervision:** Junling Zuo, Shaochuan Huo.

**Writing – original draft:** Yudi Song, Haoming Huang, Sudan Wang.

**Writing – review & editing:** Haoming Huang, Junling Zuo, Shaochuan Huo.
